# Temporal Trends and Predictors of Modern Contraceptive Use in Lusaka, Zambia, 2004–2011

**DOI:** 10.1155/2015/521928

**Published:** 2015-12-27

**Authors:** Nancy L. Hancock, Carla J. Chibwesha, Marie C. D. Stoner, Bellington Vwalika, Sujit D. Rathod, Margaret Phiri Kasaro, Elizabeth M. Stringer, Jeffrey S. A. Stringer, Benjamin H. Chi

**Affiliations:** ^1^Department of Obstetrics and Gynecology, University of North Carolina School of Medicine, 3009 Old Clinic Building, Campus Box 7570, Chapel Hill, NC 27599-7570, USA; ^2^Centre for Infectious Disease Research in Zambia, 5032 Great North Road, P.O. Box 34681, 10101 Lusaka, Zambia; ^3^Department of Epidemiology, University of North Carolina School of Public Health, 2101 McGavran-Greenberg Hall, CB No. 7435, Chapel Hill, NC 27599-7435, USA; ^4^Department of Obstetrics and Gynecology, University Teaching Hospital, P.O. Box 50110, Lusaka, Zambia; ^5^Department of Population Health, London School of Hygiene and Tropical Medicine, Keppel Street, London WC1E7HT, UK

## Abstract

*Introduction*. Although increasing access to family planning has been an important part of the global development agenda, millions of women continue to face unmet need for contraception.* Materials and Methods*. We analyzed data from a repeated cross-sectional community survey conducted in Lusaka, Zambia, over an eight-year period. We described prevalence of modern contraceptive use, including long-acting reversible contraception (LARC), among female heads of household aged 16–50 years. We also identified predictors of LARC versus short-term contraceptive use among women using modern methods.* Results and Discussion*. Twelve survey rounds were completed between November 2004 and September 2011. Among 29,476 eligible respondents, 17,605 (60%) reported using modern contraception. Oral contraceptive pills remained the most popular method over time, but use of LARC increased significantly, from less than 1% in 2004 to 9% by 2011 (*p* < 0.001). Younger women (OR: 0.46, 95% CI: 0.34, 0.61) and women with lower levels of education (OR: 0.70, 95% CI: 0.56, 0.89) were less likely to report LARC use compared to women using short-term modern methods.* Conclusions*. Population-based assessments of contraceptive use over time can guide programs and policies. To achieve reproductive health equity and reduce unmet contraceptive need, future efforts to increase LARC use should focus on young women and those with less education.

## 1. Introduction

Family planning (FP) can help individuals and couples attain their desired number of children while providing benefits across a range of areas in women's reproductive health [1]. Since the 1994 International Conference on Population and Development, national governments, international agencies, and donor organizations have redoubled efforts to ensure that FP services are universally available in Sub-Saharan Africa and elsewhere [2, 3]. In 2012 alone, it was estimated that 218 million pregnancies were prevented. In that same year, however, over 220 million women in the developing world still had an unmet need for contraception [4].

Currently, efforts to expand FP access have focused on increasing uptake of long-acting reversible contraception (LARC) [5, 6]. Highly efficacious and safe, LARC includes both subdermal implants and intrauterine contraceptive devices (IUDs) [7]. These methods are user-independent, reversible, and discreet. They do not require recurrent visits to maintain efficacy and ensure a level of privacy [7, 8]. Monitoring use of contraceptive methods, such as LARC, is critical to ongoing efforts to expand FP services. To date, however, most assessments have relied on sporadically and inconsistently collected data or older mathematical models [9, 10]. Population-level field data are urgently needed to inform and optimize FP service implementation and policy.

## 2. Materials and Methods

We conducted a secondary analysis of a repeated cross-sectional population-based survey to describe contraceptive use among women in Lusaka, Zambia. The original survey was designed to evaluate the impact of antiretroviral therapy scale-up on population-level mortality in a setting of high HIV prevalence. The methodology and primary outcomes of the survey have been detailed elsewhere [11, 12]. Briefly, between 2004 and 2011, we conducted 12 rounds of household surveys across Lusaka District, which includes Zambia's capital city Lusaka. From each of the district's 24 clinic catchments areas, 150 households were randomly sampled, for a total of 3,600 households per survey round. Fieldworkers asked a household member to identify the household head or heads. Interviewers were instructed to preferentially select female household heads because they would be more likely to recall details around household composition and events in the past 12 months. Household heads were questioned about sociodemographic characteristics, current contraceptive use or reasons for nonuse, health-related decision-making, HIV risk perception and testing history, and physical household characteristics and assets. Interviewers and participants completed all study procedures in English, Nyanja, or Bemba. Each study respondent provided written informed consent. This study was reviewed and approved by the ethical review committees from the University of Zambia Biomedical Research Ethics Committee (Lusaka, Zambia) and the University of North Carolina (Chapel Hill, NC, USA).

Our analysis was restricted to female household heads aged 16–50 years who reported not being pregnant, not being posthysterectomized, or not being postmenopausal. Male household heads were excluded from the analysis. Women who sought to delay or avoid pregnancy were asked about the specific methods they were using. Those who were not using any method of contraception were asked the main reasons behind this decision, and all reasons from each respondent were tabulated. In our analysis, contraceptive methods were categorized as modern versus traditional methods and, among those using modern contraception, we further categorized contraceptive methods into LARC or highly effective short-term reversible methods [7]. Modern contraception was defined as oral contraceptive pills (combination or progestin only), injectables (depot medroxyprogesterone acetate or norethisterone enanthate), subdermal implant, IUD, sterilization, condoms, supply methods (diaphragm, foam, or jelly), lactational amenorrhea method (LAM), or emergency contraception. LARC included subdermal implants and IUDs while highly effective short-term reversible contraception included oral contraceptive pills and injectables. LAM was excluded from the highly effective short-term reversible contraception because its effectiveness is limited to the first six months postpartum and duration of method use was not recorded for respondents. For women who reported using more than one method, they were analyzed according to the most effective method.

Descriptive statistics were used to compare characteristics between women using modern contraceptive versus those that were not. Percentages and frequencies or means with standard deviation were calculated via Chi-square or Wilcoxon tests and reported for categorical and continuous measures, as appropriate. The percentage of women using each individual method was calculated by survey year. For women not using contraception, reasons were tabulated by survey year. Among users of highly effective modern conceptive methods, logistic regression was used to create a predictive model for the odds of LARC versus short-term contraception use (pills and injectables). We did not compare those who reported LARC with those who reported traditional or no method because we wanted to understand differences between those using highly effective modern contraception. All covariates with a *p* value <0.05 were kept in the model using backwards stepwise regression. Survey year remained in the model regardless of *p* value. Prevalence trends over time were calculated using linear regression and included chronological time in years as the independent variable. Statistical analyses were conducted with Stata 13.1 (StataCorp, College Station, TX) and figures were adjusted for the complex sampling design.

## 3. Results and Discussion

**Figure 1:**
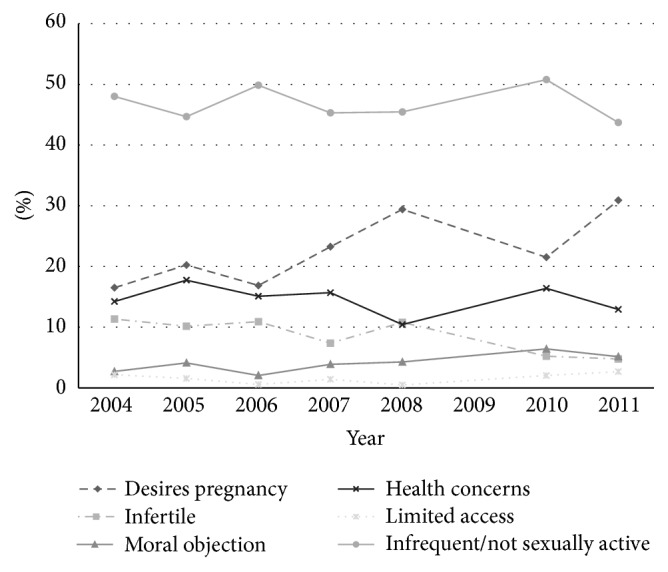
Reasons for nonuse of modern contraception among 16–50-year-old nonpregnant female heads of household in Lusaka District, Zambia, 2004–2011.

Between November 2004 and September 2011, a total of 12 survey rounds were completed across Lusaka District. Of the 43,200 heads of households who participated, 37,141 (86%) were female. Among these, 7,665 women were excluded for the following reasons: being outside the 16–50-year age range (*n* = 2,963), report of pregnancy at time of survey (*n* = 3,511), and report of prior hysterectomy or onset of menopause (*n* = 1,191). The trend in pregnancy was nonlinear, with a low of 7% in 2004 and a high of 11% in 2008. Among the 29,476 respondents who were eligible for this analysis, 17,605 (60%) reported using modern contraception (Table 1). Women using modern contraception were more likely to be under 30 years of age, be married, and have at least one child. They were also more likely to report prior HIV voluntary counseling and testing and perceive themselves at risk for HIV. Over the survey period, infrequent sex was the most frequently reported reason for not using modern contraception (45.9%), followed by desire for pregnancy (23.4%) and health concerns (14.9%) (Figure 1).

**Figure 2:**
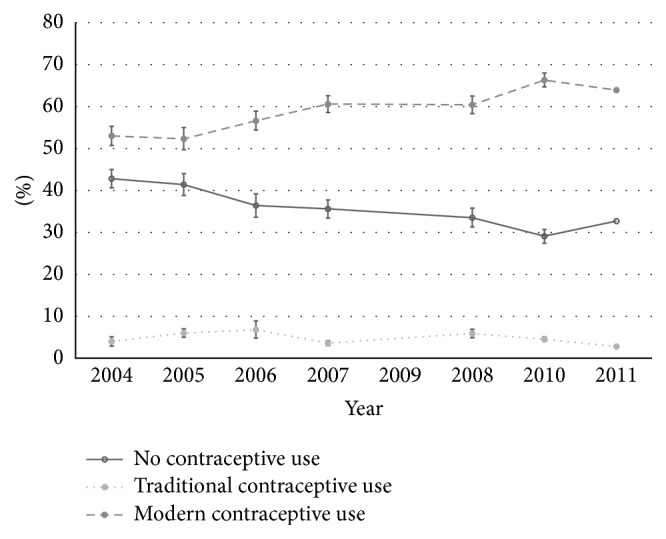
Trends in contraceptive use among 16–50-year-old nonpregnant female heads of household in Lusaka District, Zambia, 2004–2011.

**Figure 3:**
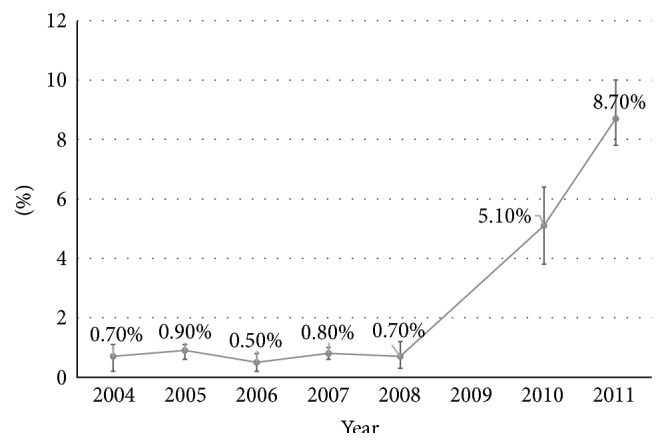
Trends in long-acting reversible contraceptive use among 16–50-year-old nonpregnant female heads of household in Lusaka District, Zambia, 2004–2011.

Reported modern contraceptive use increased significantly between 2004 and 2011 (Figure 2). In 2004, 53% of respondents endorsed using modern contraception with oral contraceptive pills being the most popular method (28%) followed by LAM (18%) and injectables (10%). By 2011, 64% of respondents reported using modern contraception. Oral contraceptive pills remained the most popular method (22%), while the frequency of injectable use increased (19%) and LAM use decreased (6%). Condom use also increased over the survey period, from 4% to 7%. LARC use was reported by less than 1% of respondents in 2004 but had increased to 9% by 2011. Significant positive changes were observed for injectable, condom, and LARC use (Table 2). The greatest increases in LARC use were observed in the final two years of the survey (Figure 3).

Among respondents endorsing modern contraceptive use, 12,964 women reported highly effective reversible contraception use (74%, Table 3). Of these, 12,098 (93%) reported use of a short-term method (pills or injectables) and 866 (7%) reported use of LARC. Factors associated with LARC use included having at least one child, having a higher socioeconomic status, and participating in the survey in 2010 and 2011. Younger women and those with less education were less likely to report LARC use. Marital status, religion, and household decision maker were not predictive of highly effective reversible contraception use (Table 3).

In this citywide repeated cross-sectional survey, we observed significant increases in modern contraceptive use generally and LARC use specifically over an eight-year period. Predictors of LARC use included older age, higher socioeconomic status, and survey year. Our findings suggest that programmatic efforts were highly successful in expanding FP services across Lusaka from 2004 to 2011, particularly in their promotion of LARC prior to 2010-2011. These population-level results are reassuring and consistent with increases in contraception uptake observed within specific health facilities [13].

We noted increased use of modern contraception over the observation period, a trend consistent with population-based surveys in Zambia. In the 2007 Zambia Demographic and Health Survey (DHS), for example, 25% of women were using a modern method, with pills, injectables, and LAM most commonly reported. Less than 1% reported LARC use [14]. By 2013-14, nearly one-third of female respondents reported using modern contraception, with injectables, pills, and subdermal implants being the most popular methods. More than 5% reported LARC use countrywide, but condom use remained steady at 4% [15]. Similar results have been shown in East Africa [10]. We observed much higher modern contraceptive use, from a baseline prevalence of 53% in 2004 to 64% in 2011. This is likely due to greater resources and higher concentration of FP initiatives in the nation's capital city, as compared to more rural and remote parts of the country, including programs focused on expanding FP access to HIV-infected women and their partners [16–19].

Perhaps the most noteworthy finding from this secondary analysis was the increase in LARC use over the final rounds of the survey. Although this study was designed to measure impact of an HIV treatment program, rather than a specific contraceptive roll-out, we believe that the major contributor to this increased coverage was a highly successful initiative led by the Zambian Ministry of Health (MOH) and the Society for Family Health (SFH). In an effort to rapidly expand services for LARC counseling and placement, this program trained and posted dedicated health providers in over 20 public health facilities starting November 2008 [13]. Over the first 14 months, 33,609 women had initiated a long-acting contraceptive method, including subdermal implants (*n* = 22,079, 66%) and IUD (*n* = 11,530, 34%). In addition, the MOH and SFH introduced postpartum IUD insertion in Lusaka in 2009, offering an additional opportunity for LARC uptake [20]. That such a high proportion of women still reported use of these methods several years later (i.e., in 2010 and 2011) is highly encouraging and suggests that LARC use can be sustained.

We observed substantial changes in the contraceptive method mix reported by women over the eight-year period. Use of oral contraceptives and LAM decreased over time, while use of injectables and LARC increased, particularly in the final survey rounds. The monitoring of such trends is important from a program perspective and can help to ensure that contraceptive supply meets ongoing demand [21]. In places like Zambia, understanding the uptake of specific methods may also be important in light of recent controversies around hormonal contraception and HIV. Although the overall evidence remains weak, some studies have suggested that hormonal contraception may be associated with increased rates of HIV acquisition among HIV-uninfected women and accelerated disease progression among women already infected with HIV [22, 23]. Recent work has also suggested that, among HIV-infected women on efavirenz, an antiretroviral agent recommended as part of first-line HIV treatment [24], certain contraceptive methods may be rendered less effective because of drug-drug interactions [25–28]. As new evidence emerges, population-level data about FP method use can help identify individuals who may need more specific method counseling and inform broader health policies around contraception.

Our study is not without limitations. First, there were several concerns inherent to the survey itself, which was primarily designed to measure all-cause mortality at a population level. Our reliance on self-identified female heads of household may have introduced selection biases, particularly around past obstetrical history and future fertility intension. Due to the nature of the questions, responses from male household heads were excluded altogether from this secondary analysis. In addition, our sampling frame, based on the year 2000 census, may not have fully represented the increasing population of urban Lusaka [12]. Second, we relied on participant self-report for our measures of contraceptive prevalence and allowed respondents to select more than one method. We did not ask about the duration of current FP method use or previously used methods, which limited our ability to further describe FP trends. We were also unable to determine the impact of method switching, from nonuse to use, from traditional to modern methods, or between various modern methods. As such, the observed increase in LARC could reflect a favorable substitution effect or a true increase in overall use. Third, it is difficult to attribute these trends to specific strategies or initiatives within Lusaka at the time of survey. More detailed information about service utilization would have greatly enhanced this analysis, including reasons why women selected one contraceptive method over another. Fourth, we provide only limited comparisons to contemporaneous DHS surveys in Zambia (i.e., 2007, 2013-2014). We noted small but important differences in our source populations that could make such comparisons difficult. When determining reasons for noncontraceptive use, for example, marital status, pregnancy, menopause, and history of hysterectomy were considered differently between our analysis and that of the DHS [29]. Finally, we recognize that the external validity of this study may be limited because our survey was only conducted in Lusaka. While we used liberal eligibility criteria for inclusion into the parent study, contraceptive use patterns likely vary between urban and rural locations in Zambia.

## 4. Conclusions

Population-based assessments of contraceptive use, like the one described in this report, provide important information to guide FP program optimization and expansion. Ongoing monitoring of contraceptive prevalence can help program managers and policy makers match the conceptive supply to ongoing demand. Properly designed surveys can also serve as an evaluative framework for assessing new and promising interventions to increase uptake and retention within FP programs. Although the optimal timing and intensity of such assessments are yet to be determined, they are a necessary component for program improvement and should be included in coordinated efforts to achieve the post-2015 development agenda.

## Figures and Tables

**Table 1:** Demographic and socioeconomic characteristics of 16–50-year-old nonpregnant female heads of household by contraceptive status in Lusaka District, Zambia, 2004–2011.

	Women using modern contraception (*n* = 17,605)	Women *not* using modern contraception (*n* = 11,871)	*p* value
Age, %			<0.001^1^
16–24	26.9	18.8	
25–30	36.6	28.1	
31–39	28.3	29.2	
40–50	8.3	24.0	
Education, %			<0.001^1^
None	3.4	5.1	
Primary	38.9	39.2	
Secondary	57.7	55.7	
Marital status, %			<0.001^1^
Married/cohabitating	92.2	61.7	
Single/divorced/widowed	7.8	38.3	
Religion, %			0.538^1^
Christian	99.2	99.1	
Other	0.8	0.9	
Living children, mean (SD)	2.76 (0.02)	2.92 (0.03)	<0.001^2^
None	4.1%	17.2%	<0.001^1^
≥1	95.9%	82.8%	
Socioeconomic status^a^, %			0.002^1^
Low	40.5	42.2	
Medium	39.3	36.6	
High	20.2	21.2	
Household decision maker, %			<0.001^1^
Woman	61.4	72.4	
Husband/male partner	22.4	14.9	
Decision made jointly	16.0	11.7	
Other	0.3	1.0	
Voluntary counseling and testing, %			<0.001^1^
Ever	79.2	59.5	
Never	20.8	40.5	
HIV knowledge^b^, %			<0.001^1^
All questions correct	68.5	64.6	
Not all questions correct	31.5	35.4	
HIV self-risk perception, %			<0.001^1^
No risk	36.3	47.7	
At risk	50.7	37.8	
Unknown	13.0	14.5	
Survey year, %			<0.001^1^
2004	7.8	10.1	
2005	15.1	20.1	
2006	8.0	9.0	
2007	26.3	25.0	
2008	8.4	8.1	
2010	8.9	6.6	
2011	25.6	21.2	

^1^ *χ* ^2^ test.

^2^Wilcoxon test.

^a^The household wealth index was created using principal components analysis based on asset variables, electricity, energy source, type of floor, number of rooms, and water and sanitation variables similar to the demographic and health surveys.

^b^As in the 2007 Zambia Demographic and Health Survey, interviewees were considered to have comprehensive knowledge of HIV if they correctly answered five questions about HIV transmission risk and so indicated that they knew that HIV cannot be transmitted through mosquitoes, HIV cannot be transmitted by witchcraft, HIV transmission risk can be reduced through condom use, HIV transmission can be reduced by having one HIV-negative sex partner, and a healthy-looking person can have HIV.

**Table 2:** Trends in contraceptive use and specific contraceptive method use among 16–50-year-old nonpregnant female heads of household in Lusaka District, Zambia, 2004–2011.

	Survey year % (95% CI)							Linear trend coefficient, *p* value
	2004	2005	2006	2007	2008	2010	2011	
No contraceptive use	42.8% (40.6, 45.0)	41.4% (38.8, 44.0)	36.4% (33.6, 39.2)	35.6% (33.4, 37.7)	33.5% (30.8, 36.1)	29.1% (26.9, 31.4)	32.7% (31.0, 34.3)	−1.6 (−1.9, −1.2), **<0.01**
Traditional contraceptive use	4.0% (2.9, 5.1)	6.0% (5.0, 7.0)	6.8% (4.8, 8.9)	3.6% (3.0, 4.2)	5.9% (4.8, 7.0)	4.5% (3.5, 5.5)	2.8% (2.3, 3.4)	−0.4% (−0.5, −0.2), **<0.01**
Modern contraceptive use	53.0% (50.7, 55.3)	52.3% (49.7, 55.0)	56.6% (54.4, 58.9)	60.6% (58.6, 62.6)	60.4% (57.4, 63.5)	66.3% (64.2, 68.4)	63.9% (62.3, 65.6)	1.9% (1.5, 2.3), **<0.01**
Condoms	3.5% (2.5, 4.6)	4.5% (3.7, 5.3)	5.3% (4.4, 6.3)	6.2% (5.4, 7.0)	5.8% (4.7, 7.0)	7.0% (5.6, 8.5)	6.7% (5.7, 7.8)	0.4% (0.2, 0.6) **<0.01**
Lactational amenorrhea	10.3% (8.1, 12.5)	10.2% (8.9, 11.5)	10.6% (7.8, 13.4)	11.8% (10.5, 13.1)	12.5% (10.1, 14.9)	7.9% (6.4, 9.3)	6.3% (5.1, 7.4)	−0.7% (−0.9, −0.4) **<0.01**
Oral contraceptive pills^a^	27.8% (26.0, 29.6)	27.2% (25.4, 29.0)	27.8% (24.6, 31.1)	27.7% (26.2, 29.1)	27.4% (24.4, 30.3)	23.6% (20.5, 26.7)	22.8% (21.6, 24.1)	−0.8% (−1.1, − 0.5) **0.08**
Injectables	9.6% (8.2, 11.0)	8.5% (7.3, 9.8)	11.6% (9.4, 13.7)	13.7% (12.5, 14.9)	13.4% (11.1, 15.7)	21.5% (19.2, 23.8)	18.6% (17.5, 19.7)	1.6% (1.4, 1.8) **<0.01**
Long-acting reversible contraception^b^	0.7% (0.2, 1.1)	0.9% (0.6, 1.1)	0.5% (0.2, 0.8)	0.8% (0.6, 1.0)	0.7% (0.3, 1.2)	5.1% (3.8, 6.4)	8.7% (7.8, 10.0)	1.3% (1.1, 1.4) **<0.01**
Implant	0.0% (0, 0.3)	0.1% (0.0, 0.2)	0.1 % (0.0, 0.2)	0.1% (0.0, 0.2)	0.0 (0.0, 0.0)	0% (0.0, 0.0)	4.1% (3.3, 4.9)	0.6% (0.4, 0.7) <0.01
IUD	0.7 % (0.2, 1.1)	0.8% (0.5, 1.0)	0.4% (0.1, 0.7)	0.7% (0.3, 1.1)	0.7% (0.3, 1.1)	5.1% (3.8, 6.4)	4.6% (3.8, 5.3)	0.7% (0.6, 0.8) <0.01
Sterilization	0.5% (0.0, 0.9)	0.8% (0.4, 1.0)	0.6% (0.1, 1.1)	0.4% (0.2, 0.5)	0.5% (0.1, 0.8)	0.9% (0.4, 1.3)	0.6% (0.3, 0.9)	0.0% (−0.0, 0.1) **0.76**
Other	0.6% (0.0, 1.3)	0.3% (0.1, 0.5)	0.2% (0.0, 0.4)	0.1% (0.0, 0.1)	0.0% (0.0, 0.0)	0.3% (0.1, 0.5)	0.2% (0.1, 0.3)	−0.0% (−0.1, 0.1) **0.24**

^a^Oral contraceptive pills include both combination and progestin only pills.

^b^Long-acting reversible contraception includes subdermal implants and intrauterine devices.

**Table 3:** Predictors of long-acting reversible contraception use among 16–50-year-old nonpregnant female heads of household using highly effective reversible contraception in Lusaka, Zambia, 2004–2011 (*N* = 12,964)^$^.

	Women using long-acting reversible contraception^+^ (*n* = 866) Percentage	Women using highly effective short-term reversible contraception^∧^ (*n* = 12,098) Percentage	Univariable OR (95% CI)	Multivariable OR (95% CI)
Age^*∗*^				
16–24	14.6%	25.8%	0.33 (0.25, 0.45)	0.46 (0.34, 0.61)
25–30	35.3%	37.3%	0.56 (0.42, 0.74)	0.66 (0.50, 0.89)
31–39	35.7%	28.5%	0.74 (0.57, 0.96)	0.80 (0.61, 1.04)
40–50	14.4%	8.5%	1	1
Education^*∗*^				
None	2.8%	3.3%	0.70 (0.42, 1.18)	0.97 (0.57, 1.65)
Primary	27.2%	38.9%	0.58 (0.45, 0.74)	0.70 (0.56, 0.89)
Secondary	70.0%	57.8%	1	1
Marital status				
Married/cohabitating	95.0%	94.8%	1.03 (0.72, 1.48)	
Single/divorced/widowed	5.0%	5.2%	1	
Religion				
Christian	99.0%	99.2%	0.72 (0.26, 2/03)	
Other	1.0%	0.8%	1	
Living children, mean (SD)	3.04 (0.08)	2.76 (0.02)	1.11 (1.06, 1.17)	
None	1.9%	2.8%		
≥1	98.1%	97.2%		
Socioeconomic status^*∗*^				
Low	27.6%	39.3%	1	1
Medium	42.9%	40.5%	1.51 (1.20, 1.90)	1.02 (0.82, 1.25)
High	29.6%	20.2%	2.09 (1.59, 2.74)	1.42 (1.14, 1.78)
Household decision maker				
Woman	64.8%	60.2%	1	
Husband/male partner	21.2%	23.4%	0.84 (0.66, 1.07)	
Decision made jointly	13.9%	16.1%	0.80 (0.63, 1.01)	
Other	0.0%	0.03%	0.17 (0.02, 1.29)	
Survey year^*∗*^				
2004	2.0%	8.1%	0.94 (0.46, 1.92)	0.98 (0.48, 1.97)
2005	4.9%	15.2%	1.25 (0.83, 1.88)	1.27 (0.86, 1.89)
2006	1.5%	8.3%	0.70 (0.37, 1.33)	0.71 (0.38, 1.34)
2007	6.8%	26.5%	1	1
2008	2.0%	8.4%	0.94 (0.50, 1.78)	0.90 (0.48, 1.70)
2010	13.6%	8.9%	5.90 (3.96, 8.80)	5.50 (3.69, 8.35)
2011	69.2%	24.6%	10.91 (7.96, 14.95)	10.73 (7.87, 14.61)

Percentages and odds ratios adjusted for complex sampling

^$^Survey data from all years were appended into a single data set.

^*∗*^Included in multivariable model.

^+^Long-acting reversible contraception includes subdermal implants and intrauterine devices.

^∧^Highly effective short-term reversible contraception includes oral contraceptive pills and injectables. Lactational amenorrhea was excluded because duration was not recorded as part of the survey.
